# Prognostic value of lipid profile in adult hemophagocytic lymphohistiocytosis

**DOI:** 10.3389/fonc.2023.1083088

**Published:** 2023-02-21

**Authors:** Wanying Cheng, Lingling Wang, Xin Gao, Limin Duan, Yongqian Shu, Hongxia Qiu

**Affiliations:** ^1^ Department of Hematology, The First Affiliated Hospital of Nanjing Medical University, Jiangsu Province Hospital, Nanjing, China; ^2^ Department of Geriatrics, The First Affiliated Hospital of Nanjing Medical University, Jiangsu Province Hospital, Nanjing, China; ^3^ Department of Hematology, Wuxi People’s Hospital, Wuxi, China; ^4^ Department of Oncology, The First Affiliated Hospital of Nanjing Medical University, Jiangsu Province Hospital, Nanjing, China

**Keywords:** lipid profile, hemophagocytic lymphohistiocytosis, prognostic, high-density lipoprotein cholesterol, mortality

## Abstract

**Background:**

Adult secondary hemophagocytic lymphohistiocytosis (sHLH) is a rare clinical syndrome with a high mortality rate. Currently, there are no feasible prognostic factors to clinically predict untreated sHLH patients’ prognosis. Our objective was to characterize the lipid profile of adult sHLH patients and to determine the relationship with overall survival.

**Methods:**

We retrospectively analyzed 247 patients with newly diagnosed sHLH from January 2017 to January 2022 according to the HLH-2004 criteria. Multivariate Cox regression analyses and restricted cubic splines were conducted to evaluate the prognostic value of the lipid profile.

**Results:**

The median age of all patients was 52 years, and the commonest cause of sHLH in our cohort was malignancy. During a median follow-up of 88 (interquartile ranges, 22–490) days, 154 deaths occurred. The univariate analysis confirmed total cholesterol (TC) ≤ 3 mmol/L, triglycerides (TG) > 3.08 mmol/L, high-density lipoprotein cholesterol (HDL-c) ≤ 0.52 mmol/L, and low-density lipoprotein cholesterol (LDL-c) ≤ 2.17 mmol/L were associated with an inferior survival. In a multivariate model, HDL-c, hemoglobin, platelet, fibrinogen, and soluble interleukin-2 receptor were considered as independent factors. Additionally, the restricted cubic spline analyses indicated an inverse linear association between HDL-c and the risk of mortality in sHLH.

**Conclusion:**

Lipid profiles, which were low-cost and readily available promising biomarkers, were strongly associated with the overall survival in adult sHLH patients.

## Introduction

Secondary hemophagocytic lymphohistiocytosis (sHLH) is the more frequent presentation of hemophagocytic lymphohistiocytosis (HLH) in adults (1). It is a rare disease associated with rapid progression that arises from infectious, rheumatological, or neoplastic triggers. Accordingly, it is, respectively, classified as infection-associated HLH (IHLH), malignancy-associated HLH (MHLH), and autoimmune-associated HLH (AHLH) (1, 2). Persistent and atypical activation of cytotoxic CD8^+^ T cells and macrophages results in the release of inflammatory cytokines are central pathogenic mechanisms (2). Characteristic features of sHLH include unremitting fever, cytopenia, hepatosplenomegaly, coagulation dysfunction, and elevation of typical HLH biomarkers, such as high triglycerides, soluble IL-2R and ferritin level (2, 3). Inadequate clinical recognition of disease severity and prognosis often leads to therapeutic delays and high mortality rates. Therefore, it is essential to identify valuable prognostic markers of adult sHLH patients.

Generally, a lipid profile refers to direct or indirect estimates of total cholesterol (TC), triglycerides (TG), high-density lipoprotein cholesterol (HDL-c), and low-density lipoprotein cholesterol (LDL-c) (4). Previous studies have suggested that serum lipid levels can indirectly measure pro-inflammatory cytokine bioactivity, even without obesity, diabetes, and metabolic syndrome (5). Additionally, components of cholesterol metabolism are essential in response to infection by moderating the immunological response during acute septic episodes (6). Moreover, the serum cholesterol levels may reflect a metabolic response of the organism to the inflammatory condition in septic patients, independent of diet or treatment (7). Consequently, systemic inflammation is associated with the lipid profile, which increases the likelihood of using it to assess the prognosis of diseases associated with inflammation.

Serum lipid anomalies are common among sHLH patients in clinical practice. Indeed, the HLH-2004 and ASH-2009 guidelines include hypertriglyceridemia as one of the main criteria for diagnosing of HLH (8, 9). Several observational studies have reported serum lipid anomalies in HLH cases (10); however, it has never been systematically studied, nor linked to disease severity or prognosis. Here, we present the first systematic prognosis research of the serum lipid in adult sHLH patients.

## Materials and methods

### Patients

This study retrospectively analyzed 247 newly diagnosed adult sHLH patients admitted to the First Affiliated Hospital of Nanjing Medical University between January 2017 to January 2022. The diagnosis of HLH was based on the 2004 HLH Diagnostic Criteria (8). Ideally, the diagnosis of adult sHLH should be further confirmed by genetic testing to rule out late-onset primary HLH in adult. However, only part patients in this study underwent genetic testing immediately after admission. Current technology and testing costs have limited patients to perform genetic testing, particularly at the early stages of the syndrome. Moreover, primary HLH is a disease of children, and the vast majority (if not all) of HLH in adults is sHLH (11). For each patient, the diagnosis of sHLH was determined based on a comprehensive assessment of clinical, laboratory, radiographic, PET/CT and histopathological findings. The diagnostic efficacy of PET/CT in sHLH has been reported in our previous study (12, 13). Therefore, the 247 patients in this study could be considered for the diagnosis of sHLH, including 166 MHLH, 69 IHLH, and 9 AHLH. The other three cases met criteria for sHLH with unknown clinical cause, and no gene mutation related to primary HLH was detected. The study was approved by the Ethics Committee of the First Affiliated Hospital of Nanjing Medical University (ChiCTR2000032421), and informed consent was obtained from the patients. The exclusion criteria were as follows. 1) Patients under the age of 18; 2) patients with cirrhosis; and 3) patients who had taken long-term lipid-lowering medicines.

Survival status was confirmed by follow-up telephone call or hospital records. Overall survival (OS) was calculated from the day of sHLH diagnosis until the date of last follow-up or death from any cause.

### Treatment

Two hundred and forty-seven patients were included, with166 malignancy-associated HLH (MHLH) and 81 non-MHLH. Among MHLH patients, 28 received the HLH-94 or HLH-04 regimen as the initial therapy (8); 67 received the systemic combination chemotherapy such as EPOCH, CHOP, or CEOP (1); 40 received the DEP regimen (14); 11 received the glucocorticoid regimen (1); 15 received supportive treatment only, and five patients received other regimens like PD-1 inhibitors (15) or plasma exchange (16). In non-MHLH patients, 11 received HLH-94 or HLH-04 therapy, 19 received DEP therapy, 25 received glucocorticoid, 17 had anti-infective treatment only, and nine patients refused any treatment or died early. No statistically significant difference was observed in the effect of treatment regimen on TC, TG, HDL-c and LDL-c.

### Statistical analysis

Baseline characteristics are summarized as median (interquartile range) as appropriate. The Mann-Whitney U and Kruskal Wallis H tests were used to compare non-normal distribution data between two or three groups. Multiple comparisons were assessed by Tukey’s test. The pairwise correlation was calculated using Spearman’s correlation analysis (“rcorr” function of the R-package “Hmisc”). Receiver operating curve (ROC) analysis was used to calculate the optimal cut-off levels for survival. The Kaplan-Meier method was applied to calculate the median OS, and comparisons were made using the log-rank test. We used univariate and multivariate Cox regression to compute the hazard ratio (HR) and the 95% confidence interval (CI) to recognize the independent prognostic factors variable on OS. Multivariate Cox regression analysis was performed with consideration of co-linearity. Moreover, restricted cubic spline curves were used to visualize the relationship between the independent and the mortality risk using the ‘rms’ packages in the R program. All statistical analyses were performed using SPSS (version 25.0, Chicago, USA), GraphPad Prism 9.0.0 (San Diego, CA), MedCalc software (version 15, Belgium), and R software version 4.0.4 (The R Foundation for Statistical Computing, Austria), and significant statistical differences were classified as *p* < 0.05.

## Results

### Clinical characteristics of the cohort

**Figure 1:**
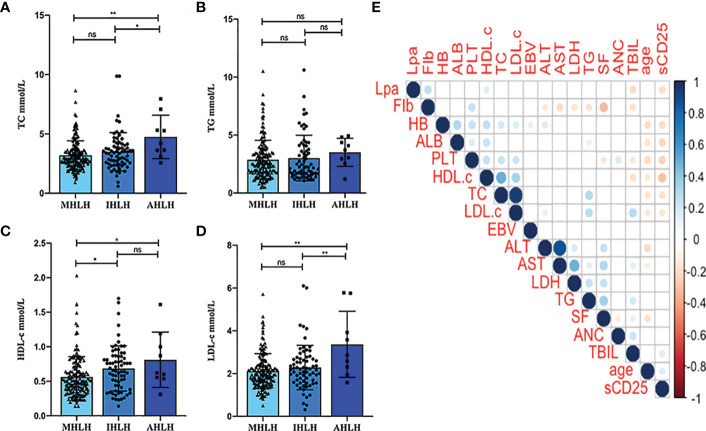
Distribution of the lipid profiles of different etiological groups and patient characteristics. **(A-D)** Comparison of TG, TC, HDL-c, and LDL-c among different etiological groups. *P < 0.05, **P < 0.01. **(E)** Pairwise correlation between the lipid profiles and age, absolute neutrophil count (ANC), hemoglobin (HB), platelets (PLT), fibrinogen (Fib), alanine transaminase (ALT), aspartate transaminase (AST), lactate dehydrogenase (LDH), Lipoprotein(a) (LPa), total bilirubin (TBIL), albumin (ALB), Epstein-Barr virus DNA copies (EBV), ferritin (SF), and soluble IL-2 receptor (sCD25). The presence or absence of bubbles indicates a statistically significant correlation. Red represents a positive correlation, while blue indicates a negative correlation. ns, Not significant, ns, P>0.05.

Detailed characteristics of the 247 participants are presented in **Table 1**. At initial diagnosis, the median age was 52 years (interquartile range 35 to 64 years). The male-to-female ratio was 1.35:1. For the entire study population, the median levels of serum TC, TG, HDL-C, and LDL-C were 3.01 mmol/L, 2.49 mmol/L, 0.54 mmol/L and 2.07 mmol/L, respectively. **Figures 1A–D** show the comparison of lipid profiles between different etiological groups. There were significant differences in the levels of TC and LDL-c between the MHLH and AHLH groups (*P* = 0.003; *P* < 0.001) and the IHLH and AHLH groups (*P* = 0.028; *P* = 0.003), while no remarkable difference was observed in TG among three groups. Serum HDL-c levels in MHLH patients was markedly lower than that in IHLH and AHLH patients (*P* = 0.014; *P* = 0.049).

**Table 1:** Baseline characteristics of 247 patients with sHLH.

Characteristics	No.	%
Age (years)	52 (35-64)	
Gender		
Male	142	57.49
Female	105	42.51
Pathogenesis		
Tumor-associated	166	67.21
Infection-associated	69	27.94
Autoimmune-associated	9	3.64
Unknown reason	3	1.21
ANC ( × 10^9^/L), median (interquartile range)	1.1 (0.68-2.56)	
HB (g/L), median (interquartile range)	82 (70-90)	
PLT ( × 10^9^/L), median (interquartile range)	44 (24-75)	
Fib (g/L), median (interquartile range)	1.39 (1.05-2.3)	
ALT (U/L), median (interquartile range)	62.4 (27.1-138.9)	
AST (U/L), median (interquartile range)	87.1 (36.4-206.2)	
ALP (U/L), median (interquartile range)	155.0 (91.9-302.6)	
LDH (U/L), median (interquartile range)	672 (414-1245)	
TBIL (umol/L), median (interquartile range)	18.1 (10.6-37.6)	
ALB (g/L), median (interquartile range)	27.6 (24.5-31.5)	
TC (mmol/L), median (interquartile range)	3.01 (2.46-3.81)	
TG (mmol/L), median (interquartile range)	2.49 (1.70-3.71)	
HDL-c (mmol/L), median (interquartile range)	0.54 (0.38-0.76)	
LDL-c (mmol/L), median (interquartile range)	2.07 (1.59-2.59)	
LPa (mg/L), median (interquartile range)	70 (21-163)	
β2-MG (mg/L), median (interquartile range)	5.61 (3.87-8.09)	
Ferritin (ug/L), median (interquartile range)	3769.9 (1506-13131)	
sCD25 (ng/L), median (interquartile range)	35741.91 (19404-43242)	
EBV infection	115	46.56
Fever	246	99.60
Splenomegaly	186	75.30
Hemophagocytic	193	78.14

ANC, absolute neutrophil count; HB, hemoglobin; PLT, platelet; Fib, fibrinogen; ALT, alanine transaminase; AST, aspartate transaminase; ALP, alkaline phosphatase; LDH, lactic dehydrogenase; TBIL, total bilirubin; ALB, albumin; TC, Total cholesterol; TG, triglyceride; HDL-c, high- density lipoprotein cholesterol; LDL-c, low-density lipoprotein cholesterol; LPa, Lipoprotein(a); β2-MG, β2- microglobulin; sCD25, soluble interleukin-2 receptor; EBV, Epstein-Barr virus.

Further analysis of the pairwise correlation between TC, TG, HDL-c, LDL-c, and baseline characteristics of patients was shown in **Figure 1E**. The TC showed a positive correlation with HB (r = 0.197; *P* = 0.002) and PLT (r = 0.096; *P* < 0.001), and was inversely associated with age (r = -0.166; *P* = 0.009) and sCD25 (r = -0.181; *P* = 0.004). TG was positively correlated with ALT (r = 0.144; *P* = 0.024), LDH (r = 0.242; *P* < 0.001), and TBIL (r = 0.207; *P* < 0.001), and inversely correlated with Fib (r = -0.170; *P* = 0.008) and ALB (r = -0.128; *P* = 0.045). HDL-c displayed positive relationships with HB (r = 0.259; *P* < 0.001), PLT (r = 0.248; *P* < 0.001), and ALB (r = 0.235; *P* < 0.001), and inverse relationships with age (r = -0.142; *P* = 0.025), TBIL (r = -186; *P* = 0.003), and sCD25 (r = -0.293; *P* < 0.001). Nevertheless, LDL-c positively correlated with HB (r = 0.168; *P* = 0.009), PLT (r = 0.217; *P* < 0.001), ALT (r = 0.136; *P* = 0.033), and TBIL (r = 0.289; *P* < 0.001), and inversely correlated with age (r = -0.149; *P* = 0.019) and sCD25 (r = -0.154; *P* = 0.015).

### Predictors of survival in sHLH patients

Among the cohorts, the median follow-up was 88 days (IQR, 22–490), with 154 observed deaths (117 for MHLH and 37 for non-MHLH). The overall 1- and 2-year survival rates were 39.1 and 33.8%, respectively. Patients with MHLH had a shorter OS than non-MHLH (*P* = 0.002). In the MHLH group, the 2-year survival rate was 24.9%, lower than 51.8% in the non-MHLH group.

**Figure 2:**
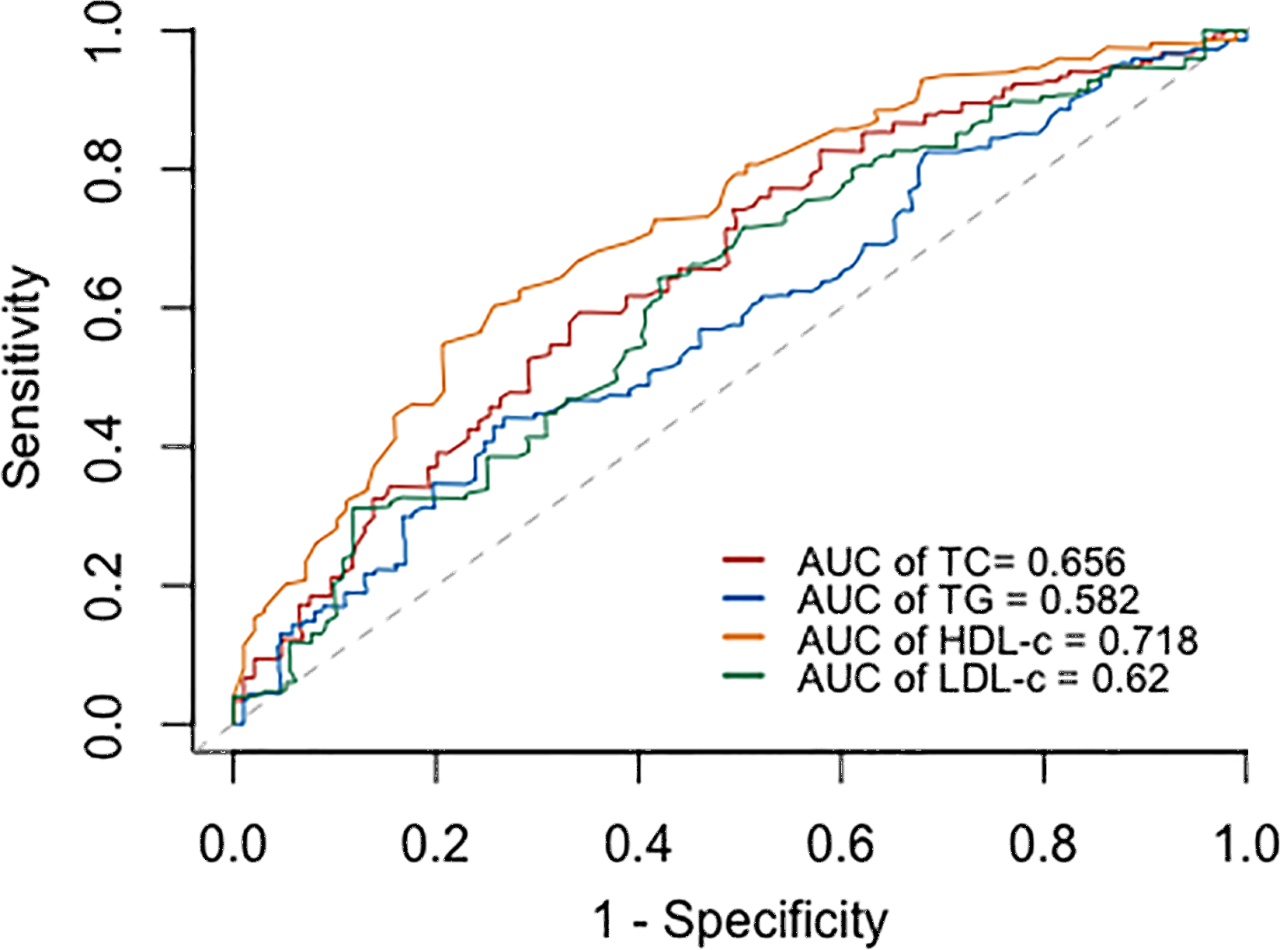
Receiver operating curve (ROC) for determining the optimal cut- off value of TC, TG, HDL-c, and LDL-c.

**Figure 3:**
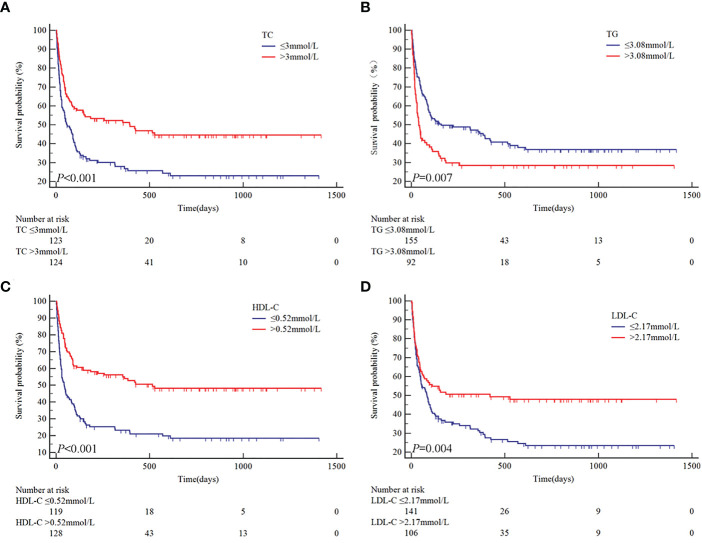
Univariate analysis of overall survival according to TC **(A)**, TG **(B)**, HDL-c **(C)**, and LDL-c **(D)**.

**Figure 4:**
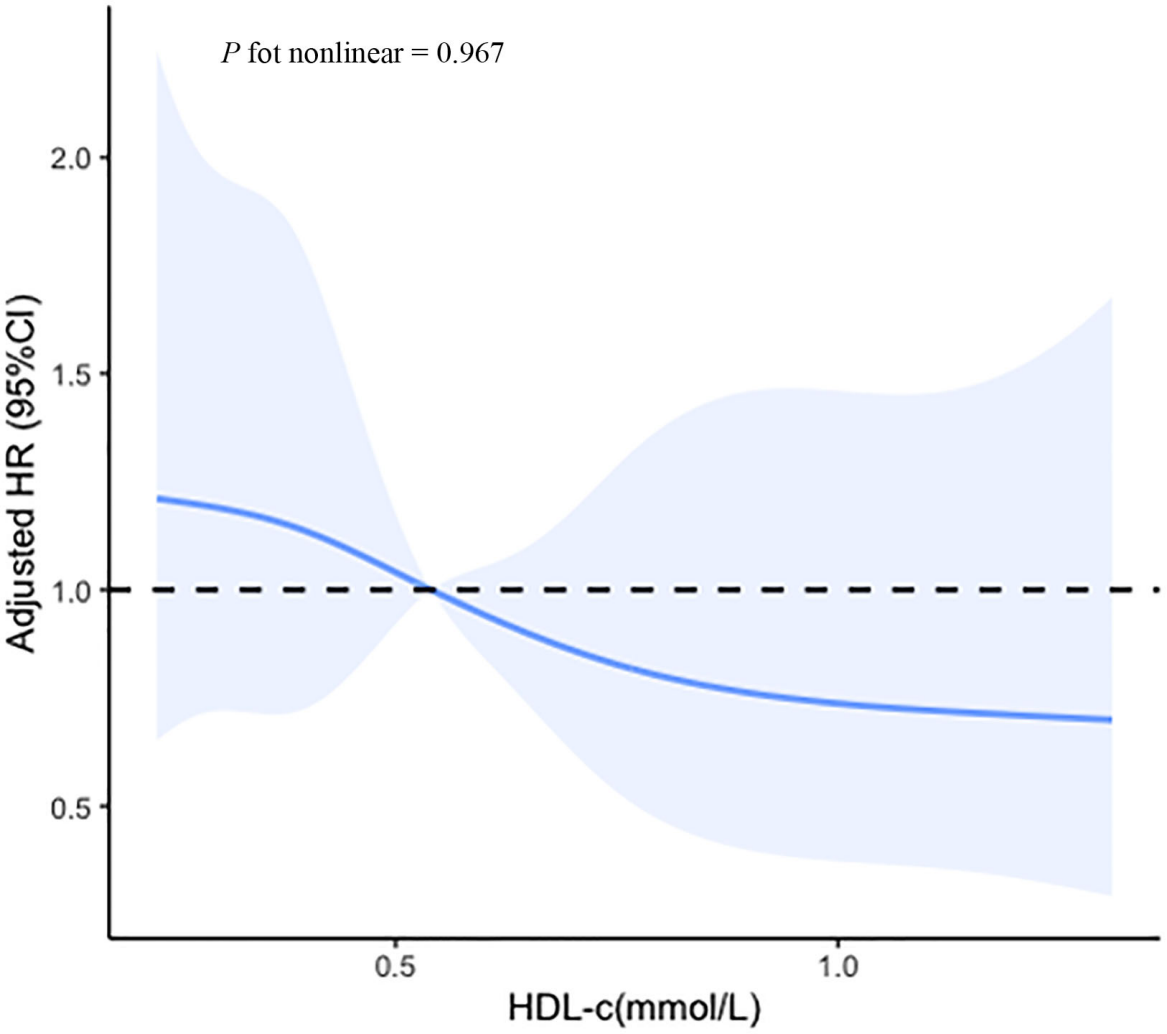
Cubic spline plot of the association between the HDL-c and the risk of mortality among sHLH patients. The solid lines and shading indicate the estimated hazard ratios and their corresponding 95% CIs. Analyses were adjusted for hemoglobin (HB), platelet (PLT), fibrinogen (Fib), and soluble IL-2 receptor (sCD25).

Based on the ROC curves, the optimal cut-off values for survival analysis for TC, TG, HDL-c, and LDL-c were 3, 3.08, 0.52, and 2.17 mmol/L (**Figure 2**), respectively. **Figure 3** shows Kaplan-Meier survival curves for TC, TG, HDL-c, and LDL-c in all sHLH patients. Compared with TC > 3 mmol/L sHLH patients, patients with TC ≤ 3 mmol/L had significantly inferior OS (median OS 60 vs. 395 days; *P* < 001). Adult sHLH patients with TG > 3.08 mmol/L had worse OS than those with TG ≤ 3.08 mmol/L (40 days vs. 160 days, *P* = 0.007). Remarkably longer survival was observed in high HDL-c and LDL-c groups (HDL-c, 515 days vs. 44 days, *P* < 0.001; LDL-c, 420 days vs. 84 days, *P* = 0.004). Furthermore, similar results were found in the MHLH group and the IHLH group (**Supplementary Figures 1**, **2**). In univariate analysis, age > 60 years, HB < 90 g/L, PLT < 100×10^9^/L, Fib ≤ 1.5 g/L, Ferritin > 10000 ug/L, sCD25 > 20000 ng/L, EBV infection, MHLH, and TG > 3.08 mmol/L were associated with an inferior outcome, whereas female, TC > 3 mmol/L, HDL-c > 0.52 mmol/L, and LDL-c > 2.17 mmol/L had better prognosis. Then the meaningful factors in univariate analysis were put into multivariate analysis. Further analysis showed that, adjusting for confounding factors, HDL-c, HB, PLT, Fib, and sCD25 were the independent prognostic factors in the multivariate Cox model (**Table 2**). In addition, restricted cubic spline (RCS) plots showed a negative linear association between the HDL-c and the risk of mortality in sHLH (**Figure 4**, *P* for nonlinearity =0.967).

**Table 2:** Univariate and multivariate analysis of predictors for overall survival in 247 patients with sHLH.

Variables (Ref)	Univariate analyses		Multivariate analyses	
	HR (95% CI)	*P*	HR (95% CI)	*P*
Female	0.64 (0.46-0.90)	**0.009**		
Age > 60 years	1.52 (1.10-2.10)	**0.011**		
ANC < 1.0×10^9^/L	1.18 (0.86-1.61)	0.316		
HB < 90 g/L	2.65 (1.70-4.14)	**< 0.001**	2.13 (1.35-3.37)	**0.001**
PLT < 100×10^9^/L	5.61 (2.08-15.17)	**0.001**	3.89 (1.42-10.61)	**0.008**
Fib ≤ 1.5 g/L	1.87 (1.34-2.61)	**< 0.001**	1.98 (1.40-2.78)	**< 0.001**
LDH ≤ 1000 U/L	1.18 (0.85-1.64)	0.333		
ALB g/L < 25 g/L	1.30 (0.92-1.83)	0.136		
Ferritin > 10000 ug/L	1.46 (1.04-2.05)	**0.028**		
sCD25 > 20000 ng/L	2.68 (1.72-4.18)	**< 0.001**	1.842 (1.12-2.96)	**0.016**
EBV infection	1.81 (1.29-2.53)	**0.001**		
Splenomegaly	0.89 (0.61-1.30)	0.552		
Hemophagocytosis	0.83 (0.55-1.24)	0.358		
MHLH (non-MHLH)	1.79 (1.23-2.59)	**0.002**		
Treatment				
Support treatment	1.00 (Ref.)			
GC±IVIg	1.46 (0.97-2.19)	0.070		
Chem±HLH94	1.08 (0.64-1.80)	0.781		
TC > 3 mmol/L	0.55 (0.40-0.76)	**< 0.001**		
TG > 3.08 mmol/L	1.55 (1.13-2.14)	**0.007**		
HDL-c > 0.52 mmol/L	0.42 (0.31-0.59)	**< 0.001**	0.55 (0.38-0.78)	**0.001**
LDL-c > 2.17 mmol/L	0.62 (0.44-0.86)	**0.004**		

ANC, absolute neutrophil count; HB, hemoglobin; PLT, platelet; Fib, fibrinogen; LDH, lactate dehydrogenase; ALB, albumin; sCD25, soluble interleukin-2 receptor; EBV, Epstein-Barr virus; MHLH malignancy-associated haemophagocytic lymphohistiocytosis; GC, glucocorticoid; IVIg, intravenous immunoglobulins; Chem, chemotherapy; HLH94, the Histiocyte Society first published international treatment protocol for HLH in 1994; TC, Total cholesterol; TG, triglyceride; HDL-c, high- density lipoprotein cholesterol; LDL-c, low-density lipoprotein cholesterol.

HR, hazards ratio; 95% CI, 95% confidence interval.

Bold: statistical significance.

### Subgroup analysis of the prognostic value of HDL-c

**Figure 5:**
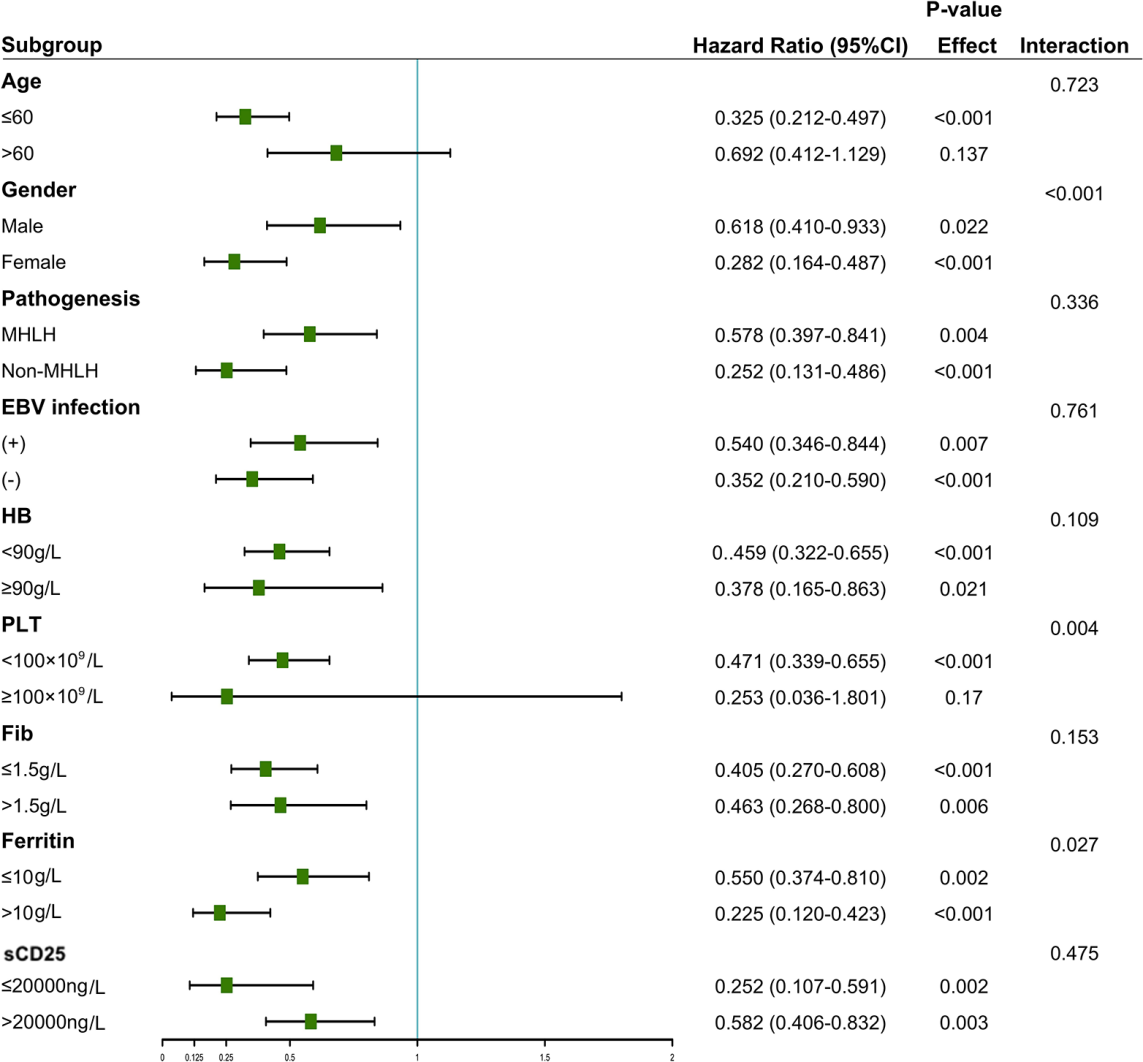
Stratified associations between HDL-c and overall survival. A Cox proportional hazards model determined the hazard ratios.

We conducted stratified analyses between HDL-c and mortality risk based on age, gender, pathogenesis, EBV infection, hemoglobin, platelet, fibrinogen, ferritin, and sCD25. **Figure 5** showed that HDL-c ≤ 0.52 mmol/L in one or two subgroups of each variable was a risk predictor for OS in sHLH patients. Although the interaction with etiological type was not statistically significant (interaction *P* = 0.336), stratified analyses suggested that the results remained consistent between MHLH and non-MHLH, indicating that irrespective of HLH pathogenesis, sHLH with high HDL-c values was associated with better OS than sHLH with low HDL-c values (*P* = 0.004; *P* < 0.001).

The pathogenesis of malignancy-associated HLH included T-cell non-Hodgkin’s lymphoma (T-NHL) (60 cases), B-cell non-Hodgkin lymphoma (B-NHL) (45 cases), leukemia (6 cases), Hodgkin’s lymphoma (2 cases), and solid tumors (2 cases). Meanwhile, there were also 50 cases of lymphoma-associated HLH diagnosed by PET in conjunction with B symptoms. Compared to T-NHL patients, B-NHL acquired a prolonger survival, although the difference did not reach statistical significance (106 days vs. 50 days, *P* = 0.058). Further subgroup analysis showed that the mortality risk of the patients with HDL-c ≤ 0.52 mmol/L was increased compared to those with HDL-c > 0.52 mmol/L in patients with T-NHL (*HR=1.89, 95%CI: 1.02-3.41*, *P* = 0.042). However, a similar trend was not found in patients with B-NHL (*P* = 0.697).

## Discussion

This study evaluated the prognostic efficiency of the lipid profile (TC, TG, HDL-c, and LDL-c) in sHLH patients before treatment for the first time. After adjusting the common prognostic factors, serum low HDL-c levels remain significantly independent risk factors for the prognosis of sHLH. Further analysis through restricted cubic spline (RCS) modeling indicated that elevated HDL-c levels had a significantly negative linear correlation with disease mortality.

Alteration in lipid profiles provides clinical information for patients’ risk evaluation and prognostic assessment. A notable observation in 247 patients with newly diagnosed sHLH was the contradictory coexistence of hypertriglyceridemia with hypocholesterolemia or low HDL-c or low HDL-c. Hypertriglyceridemia (≥ 3 mmol/L) in HLH is one of the well-known diagnostic criteria, and other studies have also shown that it may also predict a poor prognosis (17). Yu et al. (18) reported that elevated levels of triglyceride (68%) were associated with significantly lower 180-days OS rates in pediatric HLH. Consistent with these clinical observations, our results showed that an elevated triglyceride level at diagnosis correlates significantly positive with lower overall survival in adult HLH. In addition, a retrospective analysis of 227 pediatric HLH patients demonstrated that the decrease of HDL-c (99.1%) is widespread in pediatric HLH patients (10). Cascio et al. were the only authors that mentioned HDL reduction as an additional reason for the diagnosis of HLH, albeit in a footnote (19). However, data on cholesterol and cholesterol transporter levels in patients with adult sHLH are lacking, especially regarding their prognostic value. Serum lipid anomalies have also been identified in various other diseases, such as acute infections, acute fever infections with systemic inflammatory response syndrome, systemic lupus erythematosus, chronic rheumatoid arthritis, and incipient multiple organ dysfunction syndromes (5, 20–23).

In the present study, we found that increased TC, HDL-c, and LDL-c levels correlate significantly positive with overall survival. Univariate and multivariate analyses demonstrated that HDL-c was an independent prognostic factor for survival. These results are consistent with prior findings that the level of cholesterol and lipoproteins is one of the hallmarks of survival and demise in critical illness and inflammatory disorders (6, 24–26). Tanaka et al. employed 219 septic patients and found that patients with lower concentrations of lipoproteins at admission had higher 28-day mortality (27). While in multiple organ dysfunction patients, cholesterol was reported as a predictor of mortality (28). Similarly, a study revealed that reduced serum LDL-c level was an independent risk factor for the survival of patients with HBV-related acute-on-chronic liver failure (19). More recent studies have shown that low concentrations of TC, HDL-c and LDL-c were deeply associated with short-term mortality in intensive care unit septic patients (29). The strengths of this study include relatively large sample size and the first application of RCS to acquire greater statistical power and model flexibility to characterize the prognostic value of lipid profiles. Therefore, we could illustrate that lipid profiles are available for predicting the prognosis of untreated sHLH patients.

More than a decade ago, Dunham et al. reported that decreasing cholesterol levels indicated the development of infection or organ/metabolic dysfunction (30). Other investigators have reported that hypolipidemia is associated with the severity of COVID-19 (31). Thiemermann et al. (32) proposed that the magnitude of the reduction in circulating levels of HDL-c in sepsis/septic shock was positively related to the severity of the disease. Moreover, cytopenia and high levels of sCD25 are key biomarkers of HLH and are generally accepted as the severity index in sHLH (33, 34). This study built a Spearman association matrix to evaluate the relationship between lipoprotein concentrations and clinical parameters. We found that TC, HDL-c, and LDL-c have a positive association with higher hemoglobin and increased platelet count, and an inverse association with elevated sCD25, indicating that the decrease of TC, HDL-c, and LDL-c levels was positively related to the severity of the disease. Our findings suggest that serum lipid levels can indirectly measure of the severity of sHLH.

The underlying pathophysiological mechanism that changes lipid profile during the sHLH state is still unclear. One hypothesis could be that the lipid profile changes may result from cytokine release, which is central to the pathogenesis of HLH (5, 35, 36). Reportedly, decreased lipoprotein lipase activity caused by elevated TNF-α levels is accountable for increased triglyceride levels in HLH (37, 38). Several studies have revealed a strong inverse relationship between HDL-c levels and inflammatory markers, such as TNF-α, IL-1, IL-6, and INF-γ (39–42). In addition, HLH is characterized by cytokine storm and overwhelming inflammation, and at face value, these data and theories appeared to offer a more logical explanation for our present findings. Furthermore, injuries in the liver were the common symptoms of sHLH. Since liver is Since liver is an important organ for controlling cholesterol homeostasis, another possibility is that liver dysfunction might result in the disruption of cholesterol biosynthesis for controlling cholesterol homeostasis, another possibility is that liver dysfunction might result in the disruption of cholesterol biosynthesis (43). Hypocholesterolemia, low HDL-c, and low LDL-c might partially correlate with reduced cytokine-induced secretion, and/or reduced synthesis of hepatic apolipoproteins.

Our study also has several limitations: First, this is a retrospective single-center study, and potential selection bias may exist. The lack of a validation cohort might further weaken our results. Further studies, particularly a large cohort, are needed to verify our results. Second, more pro-inflammatory cytokines or inflammation markers need to be analyzed to fully establish the lipid profile’s performance characteristics. Third, the study was lacking a validation cohort.

## Conclusions

Lipid profiles, which are strongly related to the disease status, are significantly associated with the overall survival of sHLH patients. A notable observation in our study was that HDL-c was more helpful in predicting the overall survival of patients with sHLH than triglyceride, total cholesterol, and LDL-c. Further studies would be warranted.

## Data availability statement

The raw data supporting the conclusions of this article will be made available by the authors, without undue reservation.

## Ethics statement

The study was approved by the Ethics Committee of the First Affiliated Hospital of Nanjing Medical University (ChiCTR2000032421), and informed consent was obtained from the patients.

## Author contributions

WC and HQ conceived and designed the experiments. WC and XG performed the experiments. WC, LW, XG, LD and YS collected and organized the clinical materials. WC carried out data analysis. WC and HQ drafted the manuscript. All authors revised the manuscript critically and approved the final manuscript. All authors contributed to the article and approved the submitted version.
