# Supplementary material for: Prognostic value of lipid profile in adult hemophagocytic lymphohistiocytosis
Source: Front Oncol. 2023 Feb 21;13:1083088. doi: 10.3389/fonc.2023.1083088 (PMC9988898; doi:10.3389/fonc.2023.1083088)
Supplement: Supplementary file 1 [file DataSheet_1.pdf]

Supplementary Materials

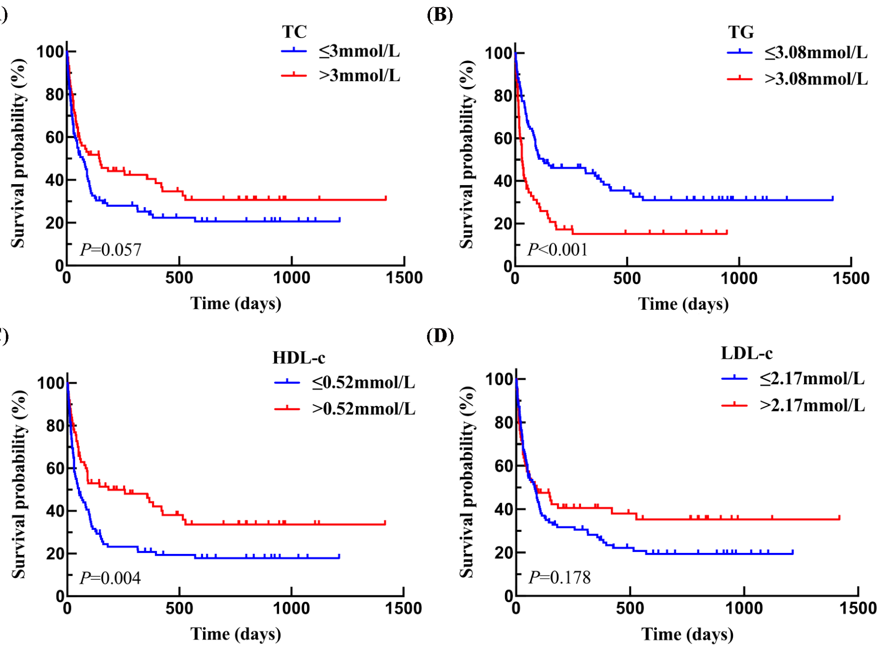

Supplementary Figure 1: Univariate analysis of overall survival according to TC (A), TG (B), HDL-c (C), and LDL-c (D) in MHLH group.

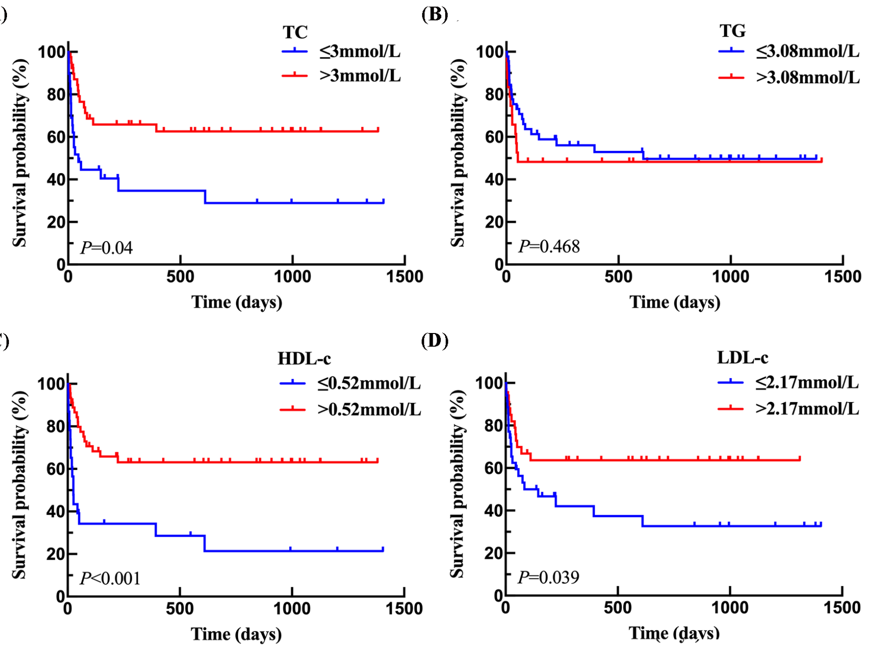

Supplementary Figure 2: Univariate analysis of overall survival according to TC (A), TG (B), HDL-c (C), and LDL-c (D) in IHLH group.
